# HIV self-testing lived experiences of female sex workers in the Garden City, Eastern Nigeria

**DOI:** 10.1177/17455057251385803

**Published:** 2025-11-11

**Authors:** Felix Emeka Anyiam, Maureen Nokuthula Sibiya, Olanrewaju Oladimeji

**Affiliations:** 1 Faculty of Health Sciences, Durban University of Technology, South Africa; 2 Vice-Chancellor and Principal’s Office, Mangosuthu University of Technology, Umlazi, South Africa; 3 Department of Epidemiology and Biostatistics, School of Public Health, Sefako Makgatho Health Sciences University, Pretoria, South Africa

**Keywords:** HIV self-testing, female sex workers, affordability, qualitative study, Nigeria

## Abstract

**Background:**

HIV self-testing (HIVST) provides a private and convenient alternative to traditional testing, addressing barriers such as stigma, fear of discrimination, and accessibility challenges. Female sex workers (FSWs) face a disproportionate burden of HIV and limited access to facility-based testing, making HIVST a critical tool for advancing Joint United Nations Programme on HIV/AIDS (UNAIDS) 2030 targets.

**Objectives:**

To explore the lived experiences of FSWs with HIVST and identify strategies to enhance its uptake and accessibility in Nigeria.

**Design:**

This was a qualitative study utilizing a phenomenological approach to explore the shared lived experiences of participants.

**Methods:**

This qualitative study utilized in-depth interviews with 15 brothel-based FSWs in Port Harcourt, Nigeria. Participants were purposively sampled to capture diverse perspectives. Thematic analysis was conducted using NVIVO-12 software to identify key themes.

**Results:**

HIVST was highly accepted due to its convenience, privacy, and autonomy. However, barriers such as cost, limited awareness, perceived inaccuracy of results, and anxiety about self-testing were reported. Participants emphasized affordability, peer support, and targeted education as key facilitators. While concerns about stigma and result interpretation were evident, participants recommended integrating HIVST into public health programs, subsidizing test kits, and expanding awareness initiatives to improve adoption.

**Conclusion:**

HIVST has significant potential to increase HIV testing rates among FSWs in Nigeria. Addressing financial, educational, and structural barriers, while leveraging peer-driven support and community engagement, is essential for optimizing HIVST uptake. These strategies align with UNAIDS’ 2030 goals and can contribute to reducing HIV-related disparities among key populations.

**Registration:**

Not applicable.

## Plain Language Summary

Exploring how female sex workers in Nigeria experience HIV self-testing and how it can be improved

This study explored the experiences and preferences of female sex workers in Nigeria regarding the use of HIV self-testing (HIVST).

*Why was the study done?*

Female sex workers face a much higher risk of HIV compared to the general population. However, many encounter stigma and fear when trying to access traditional HIV testing services at health facilities. HIV self-testing provides a way for individuals to test themselves privately and at their own convenience. This study explored the real-life experiences of FSWs with HIVST in Nigeria, to better understand what helps or prevents them from using it.

*What did the researchers do?*

The research team interviewed 15 brothel-based FSWs in Port Harcourt, Nigeria. Interviews were done face-to-face in private settings using open-ended questions to understand participants’ feelings and experiences related to HIVST. The researchers used a thematic analysis approach to identify patterns and important themes across the interviews.

*What did the researchers find?*

Participants reported that they appreciated HIVST because it allowed them privacy, control, and convenience. However, several barriers were raised, including the cost of kits, fear of test inaccuracy, emotional stress when testing alone, and concerns about stigma if others discovered their HIV testing. To make HIVST more accessible, participants suggested reducing the price of kits, using peer networks to promote self-testing, and launching wider public education campaigns. Some participants recommended integrating HIVST kits into government health programs to make them easier to access.

*What do the findings mean?*

This study highlights that HIVST is valued by FSWs but needs to be made more affordable, accessible, and better supported to increase uptake. Tailored education efforts, community-based distribution, and peer-led strategies could significantly strengthen HIV testing coverage among FSWs. These findings can help guide future policies and programs aimed at expanding HIV prevention for one of the most vulnerable populations in Nigeria.

## Introduction

HIV self-testing (HIVST) is a public health innovation that allows individuals to privately test and interpret their HIV status, addressing barriers such as stigma, discrimination, and confidentiality concerns in healthcare settings.^1^ The importance of HIVST is underscored by its potential to increase access to testing among populations disproportionately affected by HIV, particularly those in resource-limited settings.^1^ By empowering individuals to take control of their health, HIVST has emerged as a critical tool in advancing Joint United Nations Programme on HIV/AIDS (UNAIDS) 2030 goals of broad HIV testing access, ensuring that 95% of people living with HIV know their status, receive treatment, and achieve viral suppression, ultimately contributing to ending AIDS as a public health threat.^2^

Female sex workers (FSWs) are recognized as a key population in the HIV epidemic due to their heightened vulnerability and disproportionate HIV burden. Globally, the prevalence of HIV among sex workers is estimated to be approximately 3.8%, significantly higher than the global prevalence among the general adult population (0.8%).^3^ In Nigeria, where the HIV epidemic remains a significant public health challenge, the prevalence of HIV among FSWs is estimated at 15.5% (95% CI: 14.3%–16.4%), based on pooled data from the Integrated Biological and Behavioural Surveillance Survey, 2020–2021, conducted across 12 states.^4^ In Port Harcourt, Rivers State, where the present study was conducted, the HIV prevalence among FSWs was reported to be 14.5%, highlighting the critical need for context-specific, targeted interventions to address this vulnerable population.^5^ Despite their vulnerability, FSWs face unique barriers to accessing HIV testing services. Stigma,^6–8^ discrimination, and fear of legal repercussions remain significant deterrents, further marginalizing this population and limiting their access to timely diagnosis and treatment.^9^ These challenges underline the importance of innovative approaches like HIVST to bridge the gap in testing access for FSWs.

HIVST offers numerous advantages for FSWs, including convenience, confidentiality, and the ability to test in a private setting. Studies have demonstrated that HIVST can significantly increase testing uptake among FSWs by addressing key concerns associated with facility-based testing, such as long wait times, negative attitudes from healthcare providers, and the fear of being identified or reported.^10,11^ Furthermore, the autonomy provided by HIVST empowers individuals to make informed decisions about their health, thereby enhancing the likelihood of early diagnosis and linkage to care.^12^

Research found that FSWs in Côte d’Ivoire, Mali, and Senegal were not only willing to use HIVST themselves but also saw potential in distributing test kits to their clients and partners.^13^ This highlights a key opportunity to expand HIV testing coverage and reduce transmission risks, an aspect that remains underexplored due to several challenges. Issues such as limited awareness, affordability of self-test kits, and concerns about the accuracy of results and linkage to care continue to hinder widespread adoption.^14–16^ Moreover, FSWs often have limited access to comprehensive information and training on the use of HIVST kits, further complicating their ability to utilize this tool effectively.^14^ Addressing these barriers requires a comprehensive understanding of the lived experiences of FSWs with HIVST, which is critical for designing interventions that are both culturally and contextually appropriate.

Existing research on HIVST in Nigeria has largely focused on other populations with a focus on the quantitative impact of self-testing on HIV outcomes.^17–19^ While these studies provide valuable insights, there remains a significant gap in understanding the personal narratives, challenges, and preferences of FSWs who engage with HIVST. In the Nigerian context, where FSWs are disproportionately affected by HIV and face unique sociocultural and structural barriers, exploring their lived experiences is essential for informing effective HIVST policies and programs.^14^ This study aims to fill this gap by exploring the lived experiences of FSWs with HIVST in Nigeria. Understanding the lived experiences of FSWs with HIVST can provide a foundation for the development of strategies that prioritize their unique needs and preferences.

## Research methods and design

### Study design

This was a qualitative study. A phenomenological approach was used to explore the shared meaning derived from the lived experiences of individuals, offering insights into their perceptions and understanding of a particular phenomenon.^20^ This study explored participants’ experiences with HIVST broadly, without restricting participation to users of a specific self-testing modality (oral-fluid-based or blood-based). Participants discussed their perceptions of HIVST generally, based on their personal experiences or knowledge, regardless of the type of test used. Additionally, the study was conducted and reported in accordance with the Consolidated Criteria for Reporting Qualitative Research guidelines to ensure comprehensive and transparent reporting.^21^

### Setting

**Figure 1.**
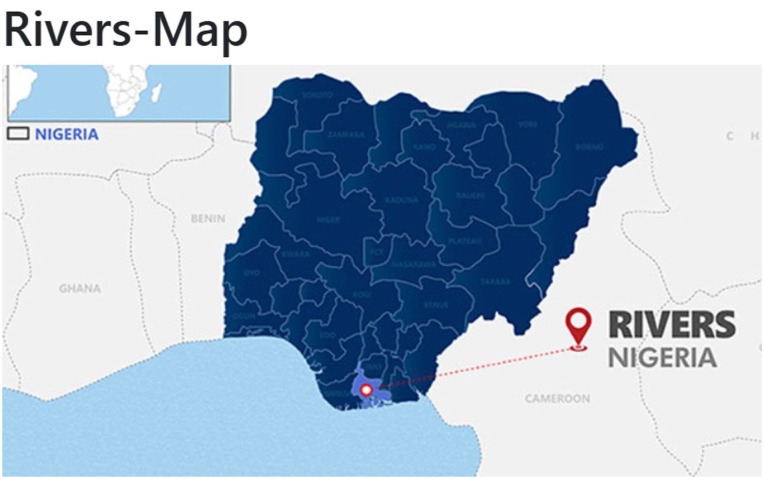
Map of Nigeria with a focus on Rivers State.^25^

The study was conducted in Port Harcourt, Rivers State, Nigeria. Port Harcourt, popularly known as the Garden City, is located in the Niger Delta region along the Bonny River. It is the largest city in Rivers State, with a population estimated at 1.86 million as of 2016.^22^ The city serves as a major industrial hub, hosting multinational firms that contribute significantly to the gross domestic product and foreign exchange revenue from crude oil. Port Harcourt attracts both street and brothel-based FSWs, with Rivers State hosting 393 identified hotspots for FSW activities.^23^ A map showing the location of Rivers State is presented in Figure 1. In this study, brothels were defined as organized establishments where sex workers live and/or work, providing sexual services to clients in exchange for money or goods, typically operating within a shared physical environment managed by brothel owners or gatekeepers.^24^

### Study population, sample size, and sampling strategy

This study targeted brothel-based FSWs aged 18–49 years who had been engaged in sex work for at least 1 year. In this study, an “FSW” was defined as an adult individual who identified as female and who regularly or occasionally received money or goods in exchange for consensual sexual services. As sex work is understood to be the consensual sale of sex between adults, individuals under the age of 18 years were not eligible to participate. This inclusive definition recognizes gender identity and aims to respect and capture the lived experiences of all individuals identifying as female within the sex work community.^26^

Inclusion criteria required that participants be identified as FSWs aged between 18 and 49 years, have engaged in sex work for at least 1 year, and had engaged in sexual intercourse within the 60 days preceding the interview, which is a recall period chosen to minimize memory decay and enhance the accuracy of self-reported behaviors. Prior research has demonstrated that shorter recall periods (3 months or less) improve the reliability of such data.^27^ In addition, only those who were available and willing to participate in face-to-face interviews and who provided voluntary written informed consent were enrolled in the study.

Exclusion criteria included individuals under the age of 18 years, who have less than 1 year of experience in sex work, and/or facing extreme threats from their sponsors (i.e. brothel managers, pimps, or individuals exerting control over their work and living conditions), which could compromise their ability to participate safely or freely in the research.

The sample size was determined based on data saturation principles, with 15 in-depth interviews conducted until no new themes or insights emerged.

The sample size was determined based on data saturation principles, with 15 in-depth interviews conducted until no new themes or insights emerged.^28^ Previous studies indicate that the proposed sample size is sufficient to reach saturation,^29,30^ and in this study, the final sample size was determined by the point at which saturation was achieved.

A purposive sampling strategy was employed to ensure a diverse representation of lived experiences, supplemented by snowball sampling to enhance recruitment through trusted peer networks. Participants were initially approached face-to-face by a trained research assistant at selected brothels, where the research team member provided information about the study, explained the voluntary nature of participation, answered any initial questions, and invited eligible individuals to participate following informed consent procedures.

In the snowball sampling phase, initial participants (primary contacts) discreetly leveraged their trusted social networks within the brothel environment to identify and refer other eligible FSWs who met the inclusion criteria and were willing to participate. This approach enhanced confidentiality, encouraged participation among individuals who might otherwise be hesitant to volunteer, and strengthened trust throughout the recruitment process. This approach facilitated confidentiality and trust, leveraging the researchers’ established relationships with local advocacy groups.

### Data collection

Data were collected using a semi-structured interview guide, between April and May 2023, developed based on a review of relevant literature on HIVST among key populations, particularly FSWs.^12,31–34^ The guide was further refined through expert consultations with public health researchers and professionals specializing in HIV prevention and qualitative research methods. A pilot test was conducted with a small subset of participants to ensure clarity, cultural appropriateness, and effectiveness in eliciting meaningful responses.

The interview guide was adapted from the World Health Organization HIV Self-Testing and Partner Notification Monitoring and Evaluation Framework (2018), a validated tool that outlines standard indicators and thematic domains related to HIVST, including awareness, acceptability, feasibility, and linkage to care. To further strengthen the content validity and contextual relevance of the guide, it was refined through expert consultations with public health researchers and professionals specializing in HIV prevention and qualitative research methods.^35^

In addition to collecting demographic data through a preliminary questionnaire, the interviews explored participants’ lived experiences with HIVST, focusing on emotions, perceptions, and behavioral responses. Probing questions encouraged participants to reflect on: their initial reactions to self-testing, their confidence in using HIVST kits, their concerns about privacy and result interpretation, and their preferences for support mechanisms, such as peer or healthcare provider assistance.

Interviews were conducted in a private setting within brothels, ensuring confidentiality and participant comfort. No one else was present during the interviews apart from the participants and the trained qualitative researcher. A trained male qualitative researcher, experienced in working with key populations, facilitated the discussions, using active listening techniques and empathetic engagement to foster a trusting environment. Each session lasted between 30 and 60 min, and all interviews were audio-recorded with participants’ written consent. To ensure ethical compliance, COVID-19 safety protocols were strictly followed.

At the time of the study, the typical retail price of HIVST kits in Nigeria ranged between 1600–2500 naira (approximately $2–$4 USD), depending on the brand and point of sale.

### Data analysis

Qualitative data were analyzed using thematic analysis guided by an inductive approach to identify recurring themes and patterns emerging from participants’ narratives.^36^ Audio recordings of the interviews were transcribed verbatim to ensure accuracy and maintain the richness of the participants’ lived experiences. All interviews were conducted in English, which participants were proficient in and preferred for the interview process.

Transcriptions were reviewed multiple times to enhance familiarity with the data and ensure the credibility of interpretations. Any unclear portions were revisited through repeated listening, and discrepancies were resolved through team discussions.

Although the semi-structured interview guide provided broad areas of inquiry, the thematic analysis was conducted independently of the guide’s topics. Initial codes and themes were developed inductively from the raw data through line-by-line coding, allowing new patterns and insights to emerge based on participants’ narratives rather than being constrained by the interview structure.

Two independent coders conducted the initial line-by-line coding of transcripts. Coding discrepancies were resolved through discussion and consensus between the coders to ensure the reliability of the analysis.

NVIVO-12 software (QSR International)^37^ was used for data organization and theme development, allowing for systematic coding and retrieval of relevant excerpts. The analysis followed a structured process in which initial codes were assigned to meaningful units of text, and similar codes were grouped into sub-themes. These sub-themes were further refined and merged into broader themes that captured the key insights from the data. To provide context and depth, themes were illustrated with direct participant quotes, ensuring that the voices of the study population were authentically represented.

### Trustworthiness

Trustworthiness was ensured through credibility, transferability, confirmability, and dependability, with specific measures taken to enhance the rigor of the study. Credibility was achieved through methodological triangulation, including verbatim transcription, field notes, and peer debriefing sessions. The interviewer, a trained male qualitative researcher in HIV prevention, conducted all interviews and reviewed transcripts against recordings to ensure accuracy.^38^ Transferability was supported by detailed descriptions of the study setting, participant characteristics, and sampling methods, allowing for the application of findings in similar contexts. The research team’s prior experience working with key populations ensured that interpretations aligned with participants’ lived realities.^39^ Confirmability was maintained through an audit trail, tracking coding decisions, and theme development. Reflexivity was practiced through a research journal documenting potential biases. Member checking was conducted, allowing selected participants to review transcripts for accuracy.^40^ Dependability was established through inter-coder reliability checks, where a second researcher reviewed coded transcripts. Supervisory reviews and iterative refinements of interview questions ensured consistency and rigor in data collection and analysis.^41^

## Results

### Participant characteristics

**Figure 2.**
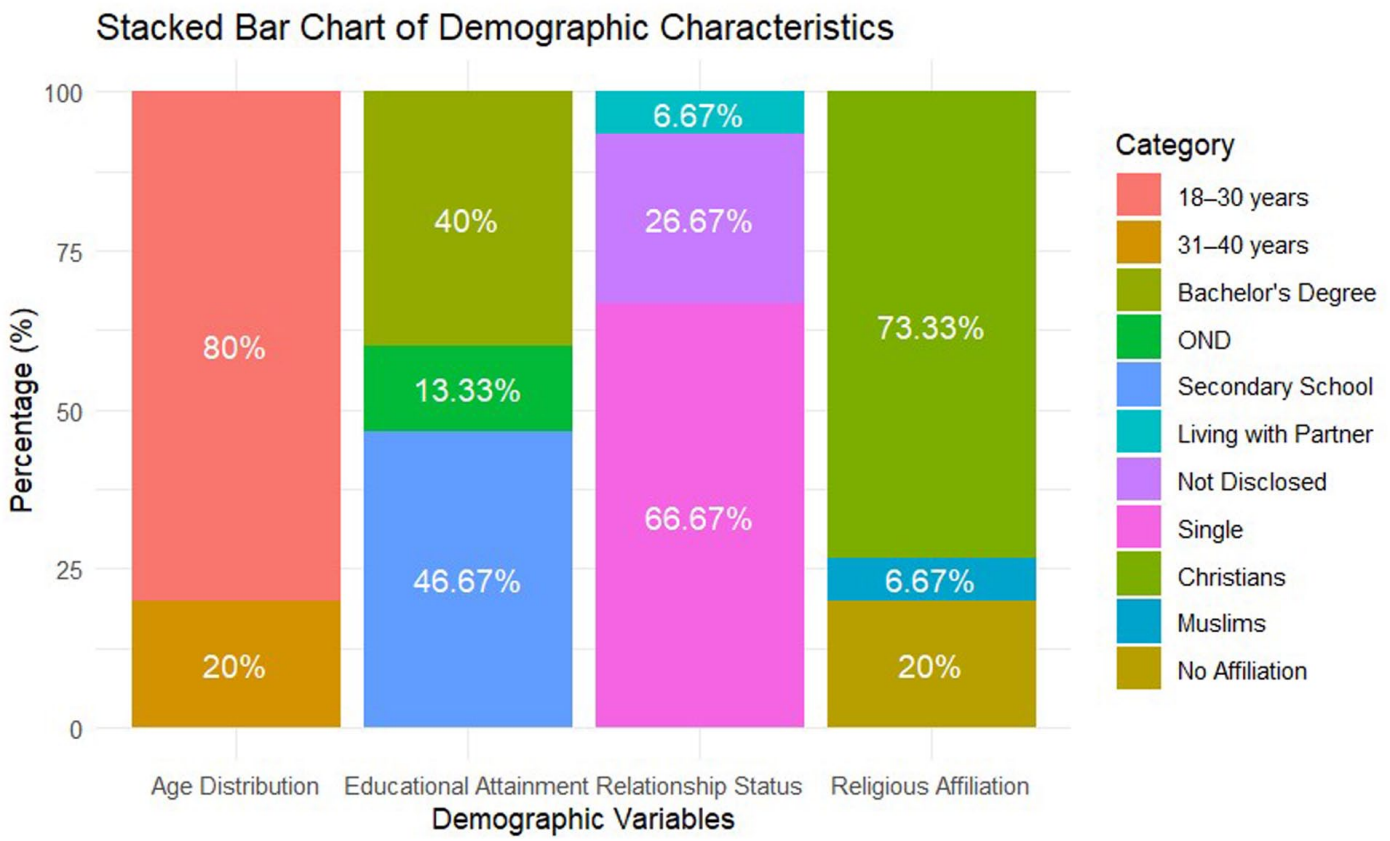
Distribution of age, educational attainment, relationship status, and religious affiliation among study participants. *Source*: Created by Authors.

Of the 15 brothel-based FSWs invited to participate, all 15 consented and completed the interviews. Interviews lasted between 30 and 60 min. All participants made significant contributions to the interview answers. Participants’ demographics are detailed in Figure 2.

The majority (80%, *n* = 12) were aged 18–30 years, with the remaining 20% (*n* = 3) between 31–40 years. Educational attainment varied, with 46.67% (*n* = 7) completing secondary school, 13.33% (*n* = 2) completing an Ordinary National Diploma, and 40% (*n* = 6) holding bachelor’s degrees. Most participants (66.67%, *n* = 10) were single, while 6.67% (*n* = 1) reported living with a partner, and 26.67% (*n* = 4) chose not to disclose their relationship status. Regarding religious affiliation, 73.33% (*n* = 11) identified as Christians, 6.67% (*n* = 1) as Muslims, and 20% (*n* = 3) had no religious affiliation (Figure 2).

The study identified four key themes related to participants’ experiences with HIVST: strategies for improving HIVST, reasons for preference, barriers and concerns, and experiences with HIVST and recommendations (Table 1).

**Table 1.** Themes and sub-themes emerging from participants’ experiences with HIVST.

Themes	Sub-themes
Strategies for improving HIVST	Affordability (e.g. affordable kits, price recommendations).
	Peer support (e.g. peer-to-peer conversations).
	Awareness initiatives (e.g. education and training campaigns).
Reasons for preference for HIVST	Privacy and Confidentiality (e.g. avoiding judgment or stigma).
	Convenience and Ease of Use (e.g. ability to test at home).
	Autonomy and empowerment through self-testing.
Barriers and concerns	Perceived inaccuracy of results (e.g. preferring traditional testing).
	Anxiety associated with self-testing (e.g. fear of needles or testing alone).
	Fear of confidentiality breaches leading to stigma and discrimination.
Experiences with HIVST and recommendations	Need for better training on how to use HIVST kits.
	Incorporating HIVST into public health schemes for improved accessibility.
	Importance of rapid results and simplicity of use.

Strategies for improvement included affordability, peer support, awareness initiatives, and targeted outreach. Preferences for HIVST were driven by convenience, privacy, and autonomy. Barriers and concerns involved perceived inaccuracy, anxiety, and fear of stigma. Experiences and recommendations highlighted the need for better training, rapid results, and integrating HIVST into public health programs.

## Strategies for improving HIVST uptake

Participants identified multiple strategies to enhance the uptake of HIVST, focusing on affordability, peer-to-peer support, and increased public awareness. Participants emphasized that reducing costs, leveraging peer networks, and conducting targeted education campaigns were critical to making HIVST more accessible, particularly among individuals hesitant to seek facility-based HIV testing services.

### Affordability and accessibility

Affordability emerged as a central determinant of HIVST uptake, reflecting the economic uncertainty faced by many FSWs. For participants, the price of self-test kits was not merely a matter of preference but a structural barrier that directly influenced their willingness and ability to test. The decision to prioritize daily subsistence often outweighed preventive health behaviors, underscoring how economic vulnerability shapes health-seeking practices. This finding resonates with evidence that high out-of-pocket costs reduce adherence to preventive services among marginalized populations.

Participants consistently stressed that unless HIVST kits are made affordable or subsidized, routine use will remain unattainable for many FSWs. One participant explained:

*For me, the availability of the kit and affordability should be prioritized. Many people are afraid or reluctant to visit hospitals for HIV testing because of the stigma they might face or the time and cost involved. If HIVST kits are made affordable and readily available, people would prefer to use them privately at their convenience instead of risking the judgment, stress, or delays that come with hospital visits.* (FSW-02)

Others went further, recommending price reductions to levels below subsistence thresholds, illustrating how deeply affordability intersects with daily survival needs:

*Yes, they need to make it very affordable for everybody, like N200 naira (less than $1 USD). Many people living in brothels or low-income areas do not have enough money to spend on health services, especially for something like HIV self-testing, which they may not see as an immediate priority. If the kits were priced low, almost anyone could afford to buy one without having to sacrifice their daily needs or worry about hospital fees. Making it cheap would encourage more people to test regularly and take control of their health.* (FSW-01)

Beyond cost, accessibility was also framed as critical. Participants emphasized the importance of integrating HIVST distribution into community-based outlets, such as local clinics and outreach programs, to minimize logistical barriers. By situating kits within trusted community spaces, HIVST could become not only economically feasible but also socially acceptable and more convenient to access. Another participant explained:

*My health is my priority and that of the health care, so I feel the testing location should be very easy for people to get tested.* (FSW-13)

### Peer-to-peer support as a strategy for HIVST adoption

Peer support emerged as a critical facilitator of HIVST uptake, underscoring the central role of social networks in shaping health behaviors among FSWs. Participants highlighted that information shared by trusted peers carried more weight than formal health messages, largely because peer educators were perceived as more relatable, less judgmental, and better able to communicate in familiar language. This dynamic reflects the importance of social influence in health-seeking behaviors, where trust and shared lived experience reduce resistance to new interventions.

Participants described how peers could recognize early signs of ill health, initiate conversations, and provide emotional reassurance, thereby reducing anxiety around testing. One participant explained:

*Peer-to-peer conversations are important because we can easily detect one who might be infected by noticing certain complaints like constant headaches or sweating. When we observe these signs, the strategy is to bring the person closer in a non-threatening way, talk to them as a peer, educate them about HIV self-testing, and encourage them to take control of their health. Hearing about self-testing from someone they trust makes it easier for them to accept and act on the information without feeling judged or afraid.* (FSW-05)

Such accounts suggest that peer-led interventions could normalize HIVST within brothel communities by embedding testing in everyday social interactions. Importantly, peers were not only seen as conveyors of information but also as sources of solidarity and emotional support, helping to counteract fears of stigma or isolation. This relational dimension implies that peer-driven approaches may be uniquely positioned to build confidence, reduce misinformation, and enhance the adoption of HIVST among FSWs.

### Raising awareness and educating the public

Participants identified limited awareness and misinformation about HIVST as significant barriers to its widespread adoption. Many felt that although self-testing offers privacy and autonomy, its benefits remain underutilized because communities lack clear, accessible information. This underscores the role of health communication not only in raising awareness but also in normalizing HIVST as part of routine health practices, thereby reducing stigma.

Participants emphasized that outreach must be delivered through channels that resonate with their daily lives. Social media and radio were identified as powerful platforms capable of reaching large audiences in both urban and semi-urban areas. These avenues allow information about HIVST to be encountered casually, woven into the routines of listening to radio programs or browsing social media, which can make the subject feel less stigmatized and more approachable. One participant explained:

*They should create awareness through social media and radio stations because these are platforms that reach a lot of people easily. People often listen to the radio during their free time, whether at work or at home, and many of us are on social media every day, scrolling through updates or chatting with friends. If messages about HIV self-testing are shared through these channels, more people will learn about it naturally as part of their daily routines, making them more likely to try it without feeling targeted or stigmatized.* (FSW-01)

Beyond mass media, participants highlighted the value of interactive community sessions and live demonstrations. Such approaches were viewed as essential for ensuring that women, particularly those with limited literacy, could develop the skills and confidence to use HIVST kits correctly. This dual emphasis on broad public messaging and localized, peer-led education reflects the need for multi-layered awareness strategies to address both informational and emotional barriers to HIVST adoption.

*By creating awareness and having an educational programme about it — not just on the radio or posters, but also in person with someone who can show us how it works — we would really understand. Many women here cannot read well, so seeing and practicing together, and hearing from other women like us, would give us the confidence to use the self-test kits correctly.* (FSW-04)

## Reasons for preference for HIVST

Participants described a strong preference for HIVST, driven by the desire for privacy, convenience, and personal autonomy. HIVST was valued for providing a confidential and accessible means to manage one’s health without fear of judgment, stigma, or disclosure to others.

### Privacy and confidentiality

Privacy and confidentiality were consistently described as decisive factors shaping women’s preference for HIVST. For many participants, the possibility of testing on their own terms provided a critical sense of safety and autonomy, enabling them to manage their health without fear of exposure or judgment. This emphasis highlights how structural stigma and social surveillance within their communities constrain conventional testing pathways, making discreet alternatives particularly valued. By allowing FSWs to control who knows about their HIV status and when disclosure occurs, HIVST offers a pathway to greater self-determination in health management.

Participants stressed that confidentiality reduced their anxiety and protected them from gossip, discrimination, and potential social exclusion. One participant explained:

*I would like to use it on my own because it’s for my privacy. I don’t want anyone to know my business or to interfere in personal matters that I prefer to keep confidential. With self-testing, I can manage my health privately without the fear of people gossiping, judging me, or making assumptions about my lifestyle. It gives me control over who knows about my HIV status and when I choose to share it, if at all.* (FSW-03)

Another participant emphasized the broader social risks associated with a lack of privacy, underscoring how HIV-related stigma can impact daily life:

*It is important to me because it’s my privacy. People are not supposed to know about my HIV status or even that I am getting tested, and it should remain confidential at all times. If people in my community find out, they might start treating me differently, gossip about me, or even discriminate against me openly. That’s why having the chance to test privately through self-testing means so much — it protects me from unnecessary judgment and social stigma.* (FSW-09)

Together, these reflections indicate that HIVST is not merely a biomedical tool but also a mechanism through which FSWs can exercise agency over their health while navigating environments where stigma and confidentiality concerns often deter engagement with traditional HIV testing services.

### Convenience and ease of use

Convenience and ease of use were strong motivators for choosing HIVST. Participants repeatedly contrasted the autonomy of testing in their own space with the frustrations of facility-based testing, which often required waiting in long lines, answering intrusive questions, and facing the possibility of judgmental attitudes from healthcare workers. The ability to avoid these barriers gave participants a sense of freedom, making HIVST not only a practical tool but also a symbol of independence and control over their own health.

As one participant explained:

*It saves me the stress of going to the hospital. I don’t have to wait in a long line or deal with the crowds at the clinic, which can be very tiring and frustrating. I also don’t have to explain myself to healthcare workers or answer uncomfortable questions about why I want to get tested. With self-testing, I can do everything privately and at my own pace, without the emotional stress or judgment that often comes with facility-based testing.* (FSW-04)

Others highlighted that the convenience of self-testing also translated into a feeling of empowerment. For some, simply being able to choose the time and setting of the test reinforced their agency in health-related decision-making:

*I felt privileged that I could use it because not everyone has the opportunity or access to test themselves privately. Having the option to test at home, without needing to go through the hospital system, made me feel empowered and independent. The fact that it was so convenient, being able to do it in my own time and space, gave me a sense of control over my health that I appreciated.* (FSW-13)

These insights reveal that HIVST’s convenience is not only about reducing structural barriers such as transport costs or clinic delays, but also about affirming participants’ right to manage their health discreetly and on their own terms. The combination of logistical ease and psychological empowerment appears to make HIVST especially attractive for FSWs, whose daily realities often limit their ability to access conventional health services.

## Barriers and concerns about HIVST

While HIVST was generally well-received, participants also expressed reservations about its limitations. Concerns centered on the accuracy of test results, the anxiety associated with self-testing in isolation, and persistent fears around confidentiality. These concerns reveal the complex interplay between biomedical trust, emotional readiness, and the social environment in which testing takes place. They also highlight that while HIVST increases autonomy, it does not fully eliminate the underlying vulnerabilities faced by FSWs.

### Perceived inaccuracy of results

A common barrier was skepticism about the reliability of self-administered HIVST kits. Participants felt that testing in a health facility, under the supervision of trained professionals, provided greater assurance of accuracy. This preference reflects broader issues of health literacy and confidence in one’s ability to perform medical procedures without error. The hesitation also underscores the importance of trust in health interventions: without confidence in the reliability of results, the convenience of self-testing may be undermined.

One participant expressed this concern clearly:

*I feel like it wouldn’t give accurate results because when you test yourself, there’s a chance you might make a mistake without even knowing it. In the hospital, you know that a trained professional is handling the test properly, following all the correct steps, and using the right equipment. That gives me more confidence that the result is accurate and reliable, which is why I prefer hospital testing over self-testing.* (FSW-14)

### Anxiety and fear of testing alone

Another barrier was the emotional burden of testing in isolation. While privacy was highly valued, some participants expressed apprehension about conducting the test without professional or peer support. For these individuals, the potential for distressing results was daunting, and the absence of immediate guidance increased their fear of misinterpreting outcomes or making harmful decisions in moments of panic. This tension illustrates the dual nature of HIVST: while it empowers autonomy, it can also heighten feelings of vulnerability when individuals are left to cope alone.

One participant described this dilemma:

*I wouldn’t want to do it alone because I am afraid of needles and the whole process makes me very anxious. If I were to get a bad result while I’m alone, I wouldn’t know what to do or how to handle the emotional shock. Without someone there to support or guide me, I fear I could panic or make wrong decisions. That’s why I would prefer to have a healthcare provider around when testing, to help explain the results and tell me what steps to take next.* (FSW-11)

This perspective emphasizes the importance of pairing HIVST with psychosocial support, whether through trained peers, hotlines, or linkage-to-care services. It also suggests that awareness campaigns should not only promote the availability of HIVST but also clarify pathways for counseling and treatment, ensuring that women do not feel abandoned at the critical moment of receiving their results.

### Concerns about confidentiality breaches

Despite valuing the privacy of self-testing, participants voiced persistent fears about the unintended disclosure of their HIV status. These concerns were particularly acute in close-knit communities, where social networks are tight and news spreads quickly. The risk of others discovering that a woman is testing, regardless of the result, was viewed as potentially damaging to her reputation, relationships, and social standing. This illustrates how stigma surrounding HIV remains a powerful barrier, shaping not only whether women test but also how they perceive the safety of testing methods.

One participant expressed this apprehension:

*If people find out, they will discriminate against you, even if you are just trying to take care of your health. In our communities, once someone hears you are testing for HIV, they may start spreading rumors or treating you differently, even if you are not positive. That is why confidentiality is very important — it protects your reputation, your relationships, and your peace of mind. Without strict privacy, people may face unfair judgment and isolation.* (FSW-09)

## Experiences with HIVST and recommendations

Participants shared both positive experiences and recommendations for improving the HIVST process, with an emphasis on education, integration into public health programs, and the importance of rapid results.

### Need for training on kit usage

Participants stressed the necessity of clear, accessible training to ensure the correct usage of HIVST kits and accurate interpretation of results. For many FSWs, the main fear was not the act of testing itself, but the possibility of making mistakes that could lead to incorrect outcomes and unnecessary emotional distress. Their reflections suggest that training is not simply a technical issue but also a way of building trust in HIVST and reducing the anxiety that comes with self-administered health interventions.

One participant emphasized this point:

*You can educate people about it and do some training because many people might not know how to use the kit properly on their own. Without proper guidance, they might make mistakes or misinterpret the results, which could cause unnecessary fear or give them a false sense of security. People need to know how to use it correctly — step-by-step — so that they can trust their results and take the right actions afterward.* (FSW-01)

### Integration into public health schemes

Participants also emphasized the importance of integrating HIVST into existing public health schemes, particularly the National Health Insurance Scheme (NHIS). This suggestion reflects recognition that sustainable uptake of HIVST requires more than availability; it must also be embedded within broader health infrastructure to ensure affordability, reliability, and continuity of care. Linking HIVST to government-supported programs was seen as a way to legitimize the intervention, reduce financial barriers, and expand its reach to underserved populations.

As one participant explained:

*Providing free health schemes, like the National Health Insurance Scheme, will help raise awareness and increase uptake of HIV self-testing. When HIVST kits are included in public health programs and made free or very low-cost, it sends a message that testing is important and accessible to everyone, not just those who can afford it. It would also encourage more people to get tested regularly without worrying about financial barriers, and it would create more visibility and trust in self-testing options across different communities.* (FSW-10)

### Importance of rapid results

Participants also valued the immediacy of HIVST results, particularly in situations of heightened risk. The ability to receive a result within minutes was seen as a major advantage over conventional facility-based testing, which often involves waiting for hours or returning days later for outcomes. Rapid results provided immediate clarity and reduced the stress associated with uncertainty, allowing FSWs to make prompt decisions about their health. This emphasis reflects how timeliness in testing can directly influence prevention and care-seeking behaviors, particularly in high-risk environments where delays may carry serious consequences.

One participant highlighted how quick results eased the burden of accessing services:

*It’s fast, and you receive your result immediately, which makes a big difference compared to going to a hospital. You don’t have to wait for hours, book an appointment, or come back days later to collect your results. With self-testing, everything happens in the moment, you test yourself and know your status right away. That saves time, reduces stress, and allows you to quickly decide what steps to take next for your health.* (FSW-01)

Another participant illustrated the value of rapid testing in urgent, high-stakes situations:

*Something happened where I used to stay, two people engaged in sexual intercourse, and by mistake, the condom busted during the act. They immediately rushed to the clinic because they were scared about the possibility of HIV transmission and wanted to get tested as quickly as possible. In situations like that, having an HIV self-testing kit on hand is very important. It would allow people to act immediately, check their status on the spot, and reduce the panic and delays that come with trying to find a clinic or waiting in long lines for testing.* (FSW-01)

These accounts emphasize that speed is not merely a matter of convenience but also a form of psychological reassurance. Rapid access to results enables FSWs to reduce anxiety, respond quickly in urgent situations, and make timely decisions about seeking confirmatory testing or treatment.

## Discussion

### Summary of key findings

This study explored the lived experiences of FSWs with HIVST in Nigeria, emphasizing their preferences, barriers to adoption, and strategies to improve HIVST uptake. Participants reported both positive and negative experiences, shaped by socioeconomic challenges, cultural factors, and structural barriers within healthcare systems. Key facilitators included the perceived benefits of privacy, autonomy, and convenience associated with HIVST. Persistent barriers included cost concerns, perceived test inaccuracies, anxiety about self-testing alone, and limited accessibility. Participants also identified several strategies to enhance HIVST uptake, including promoting affordability, leveraging peer support systems, and conducting targeted education and awareness campaigns.

### Comparison with previous studies

Participants’ emphasis on the need for affordable HIVST kits aligns with global studies that highlight affordability as a critical factor influencing uptake.^18,42^ For instance, a study demonstrated that the cost-effectiveness of HIVST kits plays a pivotal role in their acceptability, particularly among economically disadvantaged individuals.^43^ Similarly, another study observed an increase in HIV testing rates among high-risk individuals when subsidized HIVST kits were made available.^44^

Peer-driven initiatives suggested by participants are also well-supported by the literature. Studies have shown that peer-led interventions are effective in promoting HIV testing among key populations.^45,46^ A study conducted in Uganda found that distributing HIVST kits through peer networks was both practical and effective, identifying more new HIV infections compared to standard methods.^47^

Education and awareness campaigns emerged as another crucial strategy. This highlights the significant impact that spreading knowledge has on changing health-seeking behaviors.^32^ Participants stressed the importance of training and sensitization programs to address misconceptions and empower individuals to use HIVST kits confidently. One study found that individuals with knowledge of HIVST were 1.76 times more likely to test for HIV in the past year.^48^ Similarly, limited knowledge about HIVST was associated with a reduced likelihood of seeking HIV care, underscoring the critical role of awareness in promoting testing and linkage to care.^49^ These efforts would not only build trust in HIVST but also encourage individuals hesitant about clinical testing to adopt self-testing methods.

Participants’ prioritization of privacy and convenience as drivers of HIVST uptake is consistent with research conducted among FSWs in Côte d’Ivoire, Mali, and Senegal, where privacy, autonomy, and convenience were key motivations for HIVST adoption.^13^ Similar findings were reported in Tanzania, where FSWs expressed enthusiasm for HIVST due to the convenience, time-saving, and confidentiality it offers.^12^ In Gaborone, Botswana, HIVST acceptability among FSWs was driven by its convenience, privacy, and perceived protection from stigma, despite barriers such as low literacy and mistrust of test accuracy.^50^ The simplicity and rapid results provided by HIVST kits further enhanced their appeal. These attributes not only empower individuals to take charge of their health but also address logistical barriers, such as transportation and time constraints, that often hinder clinic-based testing.

Despite its advantages, participants identified several barriers to HIVST adoption, including concerns about accuracy, anxiety about self-testing, and broader systemic challenges. Skepticism about the reliability of HIVST kits was a common theme. Concerns about the accuracy of HIVST results mirror findings from a study where doubts about test reliability reduced uptake among FSWs in Northwest Ethiopia.^10^ Participants’ anxiety about self-testing alone, particularly fears related to mistakes and emotional distress, resonates with findings in other studies emphasizing the need for user support to enhance confidence and proper test use.^51,52^

Structural barriers, such as limited availability and high costs, further hindered HIVST adoption. Participants emphasized the importance of integrating HIVST into public health programs to ensure accessibility. Subsidized distribution through health schemes like the NHIS was suggested as a potential solution. Addressing these systemic barriers is critical for scaling up HIVST and ensuring equitable access, particularly in resource-constrained settings.^53^

To enhance accessibility, participants advocated for integrating HIVST into existing public health programs.^54^ Subsidized or free distribution through hospitals, clinics, and community health centers was suggested as a means to address financial barriers. Leveraging community-based approaches, such as peer-led awareness campaigns, could further promote HIVST uptake.^55^ Participants emphasized the role of peers in building trust and encouraging adoption.

### Implications of findings

The findings have important implications for policy and practice. To enhance HIVST uptake among FSWs and other vulnerable populations, national HIV programs should prioritize subsidizing HIVST kits and integrating them into public health insurance schemes such as Nigeria’s NHIS. Providing free or low-cost access would remove significant economic barriers.

Peer-driven distribution strategies should be institutionalized to promote HIVST through trusted networks, leveraging the influence of peers to reduce stigma and misconceptions. Educational initiatives should accompany these efforts, focusing on building users’ knowledge, addressing misconceptions about test accuracy, and fostering confidence in self-testing procedures.

Additionally, integrating HIVST distribution into community health services would improve accessibility. Tailored, community-based awareness campaigns could target marginalized populations and encourage broader adoption of HIVST as part of routine HIV prevention strategies.

### Strengths and limitations

This qualitative study offers rich, in-depth insights into the lived experiences of FSWs with HIVST in Nigeria, addressing a critical gap in the literature. Capturing firsthand perspectives, provides a good understanding of the facilitators and barriers to HIVST adoption, contributing valuable evidence for policy and programmatic interventions. Additionally, the use of direct participant narratives strengthens the study’s credibility, ensuring that the findings reflect real-world experiences.

However, several limitations should be noted. The study focused on brothel-based FSWs in a single city, which may limit the generalizability of the findings to other geographical regions, sex work settings (e.g. street-based or independent FSWs), and sociocultural contexts. The reliance on self-reported data may have introduced recall bias or social desirability bias, as participants may have provided responses they perceived as more socially acceptable. Additionally, the exclusion of individuals with less than 1 year of sex work experience may have limited insights into the unique vulnerabilities and HIV prevention needs of new entrants into sex work. Furthermore, although the study provides rich qualitative insights, it does not quantify the prevalence of identified themes, necessitating further research with larger and more diverse FSW populations.

An additional limitation is that the study did not differentiate between different HIVST modalities (oral-fluid-based versus blood-based); participants’ experiences were discussed generally without specifying the type of self-test used. Also, data collection was conducted by a single male interviewer experienced in working with key populations, which, while providing a supportive environment for participants, introduces the possibility of interviewer bias. The interviewer’s personal traits, beliefs, and interpretations could have influenced participants’ responses and the data collected.

Despite these limitations, the findings offer critical perspectives that can inform targeted interventions, peer-support strategies, and policy recommendations to improve HIVST access and uptake among FSWs.

### Implications for future research

Future research should differentiate between HIVST modalities (oral-fluid versus blood-based) and testing supervision models (supervised versus unsupervised) to better assess their specific impacts on acceptability and uptake among FSWs. Studies should also evaluate long-term outcomes related to the integration of HIVST into national HIV prevention programs, including linkage to care and sustained usage. Furthermore, future work should explore the role of peer networks and social support systems in promoting HIVST use and sustaining testing behaviors over time among marginalized populations.

### Recommendations

To promote equitable and widespread uptake of HIVST among FSWs and other vulnerable populations, a multifaceted approach is essential. First, HIVST kits must be made affordable and accessible through subsidies, price regulation, and community-based distribution. Second, targeted awareness campaigns should enhance knowledge, build confidence, and highlight privacy protections to reduce fear and stigma. Third, peer-driven outreach should be expanded by training peer educators to guide users, dispel myths, and normalize HIVST. Fourth, access should be broadened through discreet distribution channels like pharmacies, vending machines, and online services. Fifth, HIVST must be integrated into public health programs such as the NHIS and routine sexual health services to ensure linkage to care. Finally, further research is needed to evaluate HIVST scalability for key populations, including men who have sex with men and adolescent girls, and assess long-term impacts on prevention, diagnosis, and treatment adherence.

## Conclusion

HIVST has the potential to significantly increase HIV testing rates among FSWs in Nigeria by offering convenience, privacy, and autonomy. This study highlights the critical need for public health strategies that make HIVST more affordable, accessible, and trusted among FSWs in Nigeria. Integrating peer-led initiatives and expanding subsidized HIVST distribution could significantly enhance uptake and early diagnosis within this high-risk population, thereby supporting Nigeria’s progress toward the UNAIDS 2030 goals. Future research should explore the effectiveness of differentiated HIVST delivery models and assess their impact on linkage to care and sustained HIV prevention efforts.

## Supplemental Material

sj-doc-2-whe-10.1177_17455057251385803 – Supplemental material for HIV self-testing lived experiences of female sex workers in the Garden City, Eastern Nigeria Supplemental material, sj-doc-2-whe-10.1177_17455057251385803 for HIV self-testing lived experiences of female sex workers in the Garden City, Eastern Nigeria by Felix Emeka Anyiam, Maureen Nokuthula Sibiya and Olanrewaju Oladimeji in Women's Health

sj-docx-3-whe-10.1177_17455057251385803 – Supplemental material for HIV self-testing lived experiences of female sex workers in the Garden City, Eastern Nigeria Supplemental material, sj-docx-3-whe-10.1177_17455057251385803 for HIV self-testing lived experiences of female sex workers in the Garden City, Eastern Nigeria by Felix Emeka Anyiam, Maureen Nokuthula Sibiya and Olanrewaju Oladimeji in Women's Health

sj-pdf-1-whe-10.1177_17455057251385803 – Supplemental material for HIV self-testing lived experiences of female sex workers in the Garden City, Eastern Nigeria Supplemental material, sj-pdf-1-whe-10.1177_17455057251385803 for HIV self-testing lived experiences of female sex workers in the Garden City, Eastern Nigeria by Felix Emeka Anyiam, Maureen Nokuthula Sibiya and Olanrewaju Oladimeji in Women's Health
