# Supplementary material for: HIV self-testing lived experiences of female sex workers in the Garden City, Eastern Nigeria
Source: Womens Health (Lond). 2025 Nov 11;21:17455057251385803. doi: 10.1177/17455057251385803 (PMC12605878; doi:10.1177/17455057251385803)
Supplement: sj-doc-2-whe-10.1177_17455057251385803 – Supplemental material for HIV self-testing lived experiences of female sex workers in the Garden City, Eastern Nigeria [file sj-doc-2-whe-10.1177_17455057251385803.doc]

**Supplementary File 1: Letter of information for the interview participants**


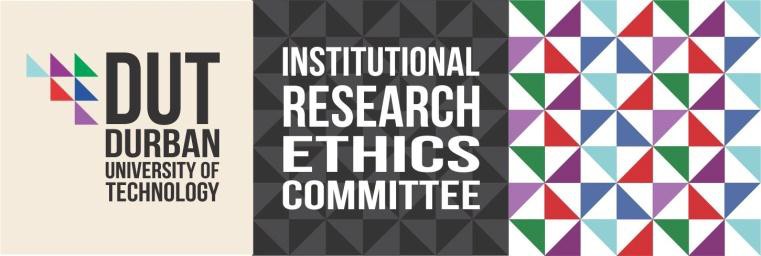


**Title of the Research Study:** Strategies to Improve the uptake of hiv self-testing among vulnerable group in Sub-Saharan Africa

**Principal Investigator/s/researcher: Felix Emeka Anyiam (**PhD: Health Sciences Candidate).

**Co-Investigator/s/supervisor/s:** Dr O. Oladimeji (PhD); Prof ‪M. N. Sibiya**‬**‬‬‬‬ (D Tech: Nursing).

**Brief Introduction of the Study:** Self-testing for HIV is an essential first step in initiating HIV prevention and treatment. Despite this, due to stigma, poor treatment by healthcare providers, and/or concerns about confidentiality, most people who are at high risk of contracting HIV or who are already infected with HIV do not get HIV testing at a high enough rate. In Sub-Saharan Africa (SSA), the perceived psychological suffering of living with HIV, financial restraints, and gender disparities continue to hinder the increasingly widespread availability of HIV testing. In order to address the first cascade of the UNAIDS 90–90–90 program, it is critical to develop and implement innovative strategies to target VPs for HIV testing in resource-constrained settings. UNAIDS' "90 90 90" targets will not be realized unless further efforts are made to improve VP access to and adoption of HIV testing.

**Purpose of the Study:** This study intends to investigate strategies/techniques for increasing HIVST uptake, which has significant policy implications and also explores sociodemographic, socioeconomic, and behavioral aspects of HIVST among SSA's most vulnerable groups by utilizing geographical clustering analysis.

Good morning,

I am a PhD student at DUT doing research for my Doctorate degree in Health Sciences.

I would like to invite you to participate in the study that seeks to identify strategies to improve the uptake of HIV Self-testing among vulnerable groups in Sub-Saharan Africa.

**Outline of the Procedures**: You will be required to fill out a questionnaire requesting for some socio-demographic information about yourself, and hereafter an in-depth interview that will ask some questions in relation to uptake of HIV Self-testing. The interview will take between 30-60 minutes.

**Risks or Discomforts to the Participant:** There are no anticipated risks or discomfort in participating in the study.

**Explain to the participant the reasons he/she may be withdraw from the Study:** The research may be terminated early in particular circumstances viz. Non-compliance, illness, adverse reactions, etc. You are entitled to withdraw from the study at any time should you wish to do so and will still continue to receive the appropriate standard of care. You can withdraw in case the research involves any form of physically invasive, or potentially harmful procedures [e.g. drug administration, needle insertion, rectal probe, pharyngeal foreign body, electrical or electromagnetic stimulation, etc.?]. You can withdraw if the data process involves blood sample collection, cutting off body flesh samples and any other fictitious act.

**Benefits:** The results of the study will provide a model and guidelines that policymakers and stakeholders can adopt for improving HIV self-testing in other to improve HIV care and treatment, and in the long term, save lives of millions of positive cases.

**Remuneration:** Your participation is completely voluntary, and you will not be compensated in any way.

**Costs of the Study**: You will not bear any costs for participating in this study.

**Confidentiality:** The information gathered will be disseminated in a way that protects the participants' privacy. Participants will not be identifiable by their names, but rather by codes. Data will be held in a secure locker, and electronic data will be kept on password-protected hardware that will be shredded after five years.

**Results dissemination:** Publications in internationally acclaimed, peer-reviewed journals e.g. World Health Bulletin. Presentations at an international conference hosted in Africa or/and diaspora.

**Research-related Injury:** Participating in the study will not result in any research-related harm.

**Storage of all electronic and hard copies including tape recordings:** The materials gathered are confidential as there are no personal identifiers. The confidential data will be preserved in a computer system that is passworded or encrypted for five years before disposal by deletion from the computer.

**Persons to contact in the Event of Any Problems or Queries:** Please contact the Supervisors: Dr O. Oladimeji, +27 622583986, [ooladimeji@hsph.harvard.edu](mailto:ooladimeji@hsph.harvard.edu) and Prof M.N. Sibiya, 031-373 2284, [nokuthulas@dut.ac.za](mailto:nokuthulas@dut.ac.za), the researcher Felix Emeka Anyiam, +2348064995462, [felixemekaanyiam@gmail.com](mailto:felixemekaanyiam@gmail.com), or the Institutional Research Ethics Administrator on 031 373 2375.

**Supplementary File 2: Consent**


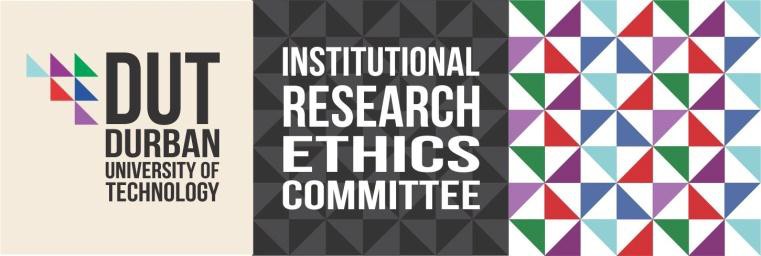


**Full Title of the Study:** Strategies to improve the uptake of HIV self-testing among vulnerable groups in Sub-Saharan Africa.

**Names of Researcher/s:** Mr Felix Emeka Anyiam.

**Statement of Agreement to Participate in the Research Study:**

 I hereby confirm that I have been informed by the researcher, Mr Felix Emeka Anyiam, about the nature, conduct, benefits and risks of this study - Research Ethics Clearance

Number: **IREC 257/22**,

 I have also received, read and understood the above written information (Participant Letter of

Information) regarding the study.

 I am aware that the results of the study, including personal details regarding my sex, age, date of birth, initials and diagnosis will be anonymously processed into a study report.

 In view of the requirements of research, I agree that the data collected during this study can be processed in a computerized system by the researcher.

 I may, at any stage, without prejudice, withdraw my consent and participation in the study.

 I have had sufficient opportunity to ask questions and (of my own free will) declare myself prepared to participate in the study.

 I understand that significant new findings developed during the course of this research which may

relate to my participation will be made available to me.

**Full Name of Participant Date Time Signature / Right**

**Thumbprint**

I, Felix Emeka Anyiam herewith confirm that the above participant has been fully

informed about the nature, conduct and risks of the above study.

Felix Emeka Anyiam

**Full Name of Researcher Date Signature**

**Full Name of Witness (If applicable) Date Signature**

**Full Name of Legal Guardian (If applicable) Date Signature**

**Supplementary File 3: Demographic data of the interview participants**

**
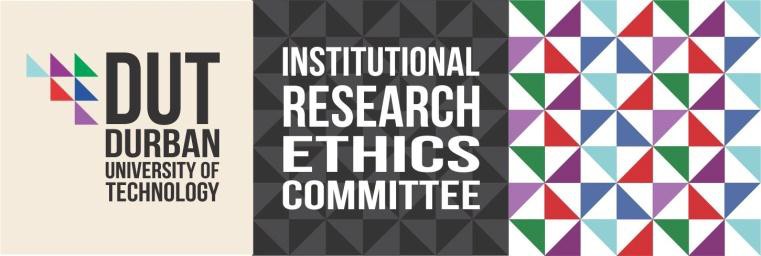
**

My name is Felix Emeka Anyiam, a PhD. in Health Sciences student at the Durban University of Technology, South Africa. I am undergoing a study that intends to investigate strategies/techniques for increasing HIVST uptake, which has significant policy implications and also explores sociodemographic, socioeconomic, and behavioral aspects of HIVST among SSA's most vulnerable groups by utilizing geographical clustering analysis.

You are required to complete this questionnaire requesting specific socio-demographic information about yourself, followed by an in-depth interview in which you will be asked several questions about HIV self-testing participation. The interview will last between 30 and 60 minutes.

**Demographic data**

Participant number:

Date: ______________________________

**How old are you (in years)** ____________

1. **Please indicate your age instead if you choose not to answer in #1 above**

| 18-30 years |  |
| --- | --- |
| 31-40 years |  |
| 41-50 years |  |
| More than 50 years |  |

1. **Which of the following best describes your religion / faith / philosophy / spirituality?** (Please check all that apply.)

- a. Christian
- b. Muslim
- c. African traditional
- d. No religious affiliation
- e. Other (please specify: ________________)
- f. I choose not to answer

1. **What is your relationship status right now?**

- 1. Single
- 2. Separated/Divorced/Widowed
- 3. Married/Common-law
- 4. Other (please specify: )
- 5. I choose not to answer

1. **What is the highest level of education you have completed?**

- a. Primary school (elementary school)
- b. Secondary school (high school)
- c. Technical or vocational
- d. Completed Bachelors degree
- e. Completed Masters degree
- f. Completed PhD
- g. Other (please specify: )
- h. I choose not to answer

1. **Over the past one year, have you engaged in any other occupation?**

- a. Yes
- b. No

1. **If yes, what has your occupation been: (Please check all that apply)**

- a. Employed or Self Employed full time work
- b. Employed or Self Employed part time work
- c. Working as a volunteer
- d. Unemployed, actively looking for work
- e. A full or part time student
- f. Not working due to disabilities
- g. Other (please specify: )
- h. I choose not to answer

**Supplementary File 4: Interview guide**

**
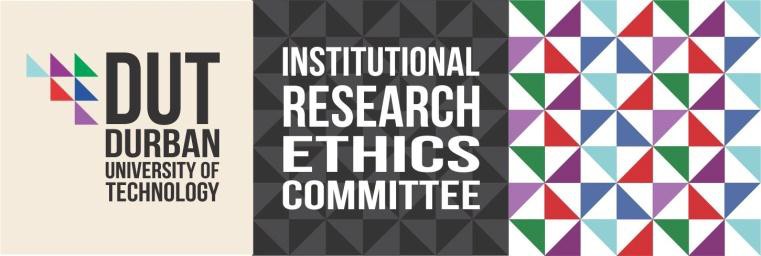
**

**Interview Questions**

1. **Experiences with HIV-testing -FOR ALL PARTICIPANTS**
2. What does the word the Human Immunodeficiency Virus (HIV) mean to you?
3. What does the word Sexually Transmitted Infection (STI) mean to you?
4. Have you ever heard of HIV Testing?
5. **If YES**, Could you describe it to me?
6. Have you been tested for HIV?

For those that said **NO**

1. What discouraged you from testing?

***Prompts***

- Have you ever faced any barriers when considering HIV testing? If so, can you share your experiences?
- Have you ever encountered any stigma or discrimination related to HIV or testing?
- Have you ever encountered a lack of access to a health facility to test?
- Does your social or support network play a role in your decision-making process regarding HIV testing?
- Are there any cultural, religious, or personal beliefs that have shaped your decision not to test for HIV?

1. If you want to test now, where would you rather go?
2. What would make it easier for you to get tested?

For those that said **YES**

1. In which location did you complete the most recent test?
2. When did that take place?
3. How would you describe the experience?
4. What did you like about it?
5. What did you dislike?
6. What motivated you to get tested?
7. **HIV self-testing (HIVST) – FOR ALL PARTICIPANTS**
8. Have you ever heard of HIV self-testing (HIVST)?
9. If so, what do you know about it? (For those that said **YES)**
10. Have you ever used an HIV self-test?
11. If so, what was your experience (For those that said **YES)**
12. If not, would you be willing to try it? (For those that said **NO)**
13. **HIVST Usage In The Past – FOR THOSE THAT SAID YES TO USING IT**
14. How did you feel about using the HIV self-test kits?
15. What are your reasons for considering using an HIV self-test?
16. Are you concerned about privacy and confidentiality when using an HIV self-test?
17. How important is privacy and confidentiality to you?
18. Are you concerned about Accessible testing locations?
19. How vital is accessible testing locations to you?
20. **Strategies To Encourage HIVST - FOR ALL PARTICIPANTS**
21. What do you think are the strategies that will encourage people to self-test?

***Prompts***

How important are the following to you:

- **Awareness and education**: Conducting public health campaigns to raise awareness about the availability, benefits, and proper usage of HIV self-testing kits, while dispelling myths and misconceptions surrounding HIV.
- **Accessibility**: Ensuring that HIV self-testing kits are easily accessible through various channels, such as pharmacies, online retailers, community centers, and healthcare facilities, at affordable prices or free of cost for those in need.
- **Confidentiality**: Promoting self-testing as a confidential and private alternative to facility-based testing, which may be particularly appealing to individuals who are hesitant to access healthcare services due to concerns about privacy or stigma.
- **Peer support and outreach**: Utilizing peer educators and community health workers to promote self-testing, share information, and provide support or guidance in using the self-testing kits.
- **Integration with existing services**: Incorporating HIV self-testing into existing health promotion programs or events, such as sexual health education sessions or workplace health initiatives.
- **Partner and social network testing**: Encouraging individuals to share self-testing kits with their partners or social networks, potentially increasing the uptake of testing among hard-to-reach populations.
- **User-friendly kits:** Ensuring that self-testing kits are easy to use, with clear instructions and support materials, such as videos or helplines, to assist users with the testing process and interpretation of results.
- **Linkages to care:** Providing clear information on how and where to access follow-up care, counseling, and treatment for individuals who test positive, ensuring they receive appropriate support and care.
- **Legal and policy support:** Advocating for policies that promote and support the use of HIV self-testing, while addressing potential barriers or legal restrictions that may hinder access to self-testing kits.

1. Do you have concerns about the affordability of HIV self-tests?
2. If the Government was not providing it for free, what is the maximum you would you be prepared to pay for such a prevention pack? (In Nigerian Naira)?
3. Would you prefer to use an HIV self-test alone or with the assistance of a healthcare provider or counselor? And Why?
4. What suggestions do you have for improving the availability of HIV self-testing in your community?
5. What suggestions do you have for improving the accessibility of HIV self-testing in your community?
6. What suggestions do you have for improving the uptake of HIV self-testing in your community?

**Thank you for your time to participate in the study.**
