# Supplementary material for: HIV self-testing lived experiences of female sex workers in the Garden City, Eastern Nigeria
Source: Womens Health (Lond). 2025 Nov 11;21:17455057251385803. doi: 10.1177/17455057251385803 (PMC12605878; doi:10.1177/17455057251385803)
Supplement: sj-docx-3-whe-10.1177_17455057251385803 – Supplemental material for HIV self-testing lived experiences of female sex workers in the Garden City, Eastern Nigeria [file sj-docx-3-whe-10.1177_17455057251385803.docx]

**Coding Tree**

**Theme 1: Strategies for Improving HIVST**

**Subtheme 1.1: Affordability**

**Codes:**

- High cost as a barrier
- Desire for price reduction or subsidization
- Suggested affordable price range for FSWs

**Subtheme 1.2: Peer Support**

**Codes:**

- Importance of peer-to-peer encouragement
- Learning through trusted peer networks
- Greater trust in peer-led initiatives over formal outreach

**Subtheme 1.3: Awareness Initiatives**

**Codes:**

- Education campaigns on correct HIVST use
- Need for community-based sensitization
- Use of mass media or group sensitization for awareness

**Theme 2: Reasons for Preference for HIVST**

**Subtheme 2.1: Privacy and Confidentiality**

**Codes:**

- Avoidance of judgment from healthcare workers
- Reduction in fear of public disclosure
- Testing in private, familiar settings

**Subtheme 2.2: Convenience and Ease of Use**

**Codes:**

- Time-saving (avoiding long queues)
- Simple instructions and user-friendly kits
- Testing at one’s own time and pace

**Subtheme 2.3: Autonomy and Empowerment**

**Codes:**

- Feeling in control of personal health decisions
- Empowerment through independent testing
- Reduced dependence on health facilities

**Theme 3: Barriers and Concerns**

**Subtheme 3.1: Perceived Inaccuracy of Results**

**Codes:**

- Distrust in results without confirmation
- Preference for professional testing at facilities
- Concerns about reading results correctly

**Subtheme 3.2: Anxiety Associated with Self-Testing**

**Codes:**

- Emotional distress while testing alone
- Fear of needles (in blood-based kits)
- Stress about potential positive results

**Subtheme 3.3: Fear of Confidentiality Breaches**

**Codes:**

- Concern about test kits being discovered
- Fear of gossip or exposure from peers or family
- Risk of stigmatization and discrimination

**Theme 4: Experiences with HIVST and Recommendations**

**Subtheme 4.1: Need for Better Training**

**Codes:**

- Calls for demonstration-based training
- Requests for clearer instructional materials
- Training on what to do post-test

**Subtheme 4.2: Incorporation into Public Health Schemes**

**Codes:**

- Distribution through NHIS or government outlets
- Integration into broader HIV prevention programs
- Making kits available at public clinics and hospitals

**Subtheme 4.3: Importance of Rapid Results and Simplicity**

**Codes:**

- Appreciation of short result turnaround
- Preference for saliva-based or less invasive methods
- Positive feedback on intuitive design
